# Current Understanding of Innate Immune Cell Dysfunction in Childhood Undernutrition

**DOI:** 10.3389/fimmu.2019.01728

**Published:** 2019-07-29

**Authors:** Claire D. Bourke, Kelsey D. J. Jones, Andrew J. Prendergast

**Affiliations:** ^1^Centre for Genomics & Child Health, Blizard Institute, Queen Mary University of London, London, United Kingdom; ^2^Zvitambo Institute for Maternal and Child Health Research, Harare, Zimbabwe; ^3^Kennedy Institute for Rheumatology, University of Oxford, Oxford, United Kingdom; ^4^Department of Paediatric Gastroenterology & Nutrition, University of Oxford NHS Foundation Trust, Oxford, United Kingdom

**Keywords:** undernutrition, malnutrition, innate immune cells, inflammation, enteropathy, infections, low- and middle-income countries (LMIC), children

## Abstract

Undernutrition affects millions of children in low- and middle-income countries (LMIC) and underlies almost half of all deaths among children under 5 years old. The growth deficits that characterize childhood undernutrition (stunting and wasting) result from simultaneous underlying defects in multiple physiological processes, and current treatment regimens do not completely normalize these pathways. Most deaths among undernourished children are due to infections, indicating that their anti-pathogen immune responses are impaired. Defects in the body's first-line-of-defense against pathogens, the innate immune system, is a plausible yet understudied pathway that could contribute to this increased infection risk. In this review, we discuss the evidence for innate immune cell dysfunction from cohort studies of childhood undernutrition in LMIC, highlighting knowledge gaps in almost all innate immune cell types. We supplement these gaps with insights from relevant experimental models and make recommendations for how human and animal studies could be improved. A better understanding of innate immune function could inform future tractable immune-targeted interventions for childhood undernutrition to reduce mortality and improve long-term health, growth and development.

## Introduction

In low- and middle-income countries (LMIC) childhood undernutrition manifests as growth deficits which can result in a child being too short for their age (stunted; height-for-age Z (HAZ) score <-2), and/or too thin for their height (wasted; weight-for-height Z (WHZ) score <-2), which are both associated with a greater risk of all-cause mortality (1). Severe acute malnutrition (SAM), the most life-threatening form of undernutrition, is defined as WHZ ≤ -3, mid-upper arm circumference (MUAC) <11.5 cm and/or bilateral pitting oedema in children between 6–59 months of age; when associated with complications, hospitalized children have a very high mortality of 10–30%. Collectively, undernutrition underlies an estimated 45% of all deaths among children under 5 years old (2). Despite significant reductions in the prevalence of undernutrition globally, stunting and wasting remain aggregated in the poorest regions of Asia and Africa (3), with sub-national foci in South Asia (3) and nearly all sub-Saharan African countries (4). At current rates of progress, few African countries are on-target to meet the Sustainable Development Goal of ending malnutrition in all its forms by 2030 (4).

Stunting and wasting are the most readily measurable indicators of undernutrition, but these anthropometric deficits result from underlying defects in multiple physiological processes (5). The most life-threatening consequence of undernutrition is an increased susceptibility to infections; the risk of infectious death increases incrementally among children with the severity of wasting and stunting (1). Children admitted to hospital with complicated SAM have a high burden of bacterial, parasitic and viral infections and mortality is driven by a range of species (6–8), highlighting that there is no single causative pathogen. After discharge from hospital, infectious morbidity and mortality persists among children recovering from SAM (7, 9, 10). One plausible interpretation is that immune defenses against infection are impaired during undernutrition and that resolution of these defects lags behind nutritional rehabilitation. Therapeutically targeting defects in the immune system could provide a novel way to reduce the burden of infections in undernourished children, since there is growing evidence that undernutrition compromises immune-mediated defenses (5). However, despite several decades of study, we still know remarkably little about the nature of immune dysfunction or its long-term health implications for the 155 million stunted and 52 million wasted children globally (11). In particular, it is not known if/how the body's first line of defense against pathogens—the innate immune system—adapts and contributes to the undernourished state (5). Restoration of impaired innate immune cell function may be a necessary yet under-studied facet of nutritional rehabilitation.

In this review we will discuss the current evidence for the role of innate immune cell dysfunction in undernutrition, focusing on children living in LMIC. We will draw on population studies and supplement knowledge gaps from existing undernutrition research with insights from experimental models. Our goal is to highlight the potential for innate immune cell dysfunction to shape clinical outcomes of undernutrition and outline how future studies could be used to improve our understanding of these pathways.

### Pathways Shaping Innate Immune Function During Undernutrition in LMIC

Undernutrition is most common among children with low dietary diversity and low energy density in the context of a marginal, monotonous diet, leading to inadequate nutrient intake, uptake and utilization. However, diet is just one component of undernutrition, during which there is simultaneous derangement of multiple physiological pathways that are key to healthy growth. These include: (1) recurrent symptomatic infections and sub-clinical pathogen carriage; (2) chronic systemic inflammation and immune activation; (3) impaired gut function, enteropathy and dysbiosis of the gut microbiome (12, 13); (4) metabolic derangement (14, 15); (5) dysregulated growth hormone axis (16, 17); and (6) multiple macro- and micronutrient deficiencies (18). Undernourished children have often also been exposed to undernutrition *in utero*: a principal predictor of postnatal growth is maternal nutritional status during pregnancy, and length and weight at birth (19). These influences combine to worsen growth and developmental outcomes in early life (20). The result is an adapted physiological state shaped by the need to prioritize current survival in the face of these challenges over future physiological potential. It is likely that immune functional capacity also adapts to the undernourished state, since immune cells can directly sense infections and microbiome components, inflammatory mediators, tissue damage, metabolites, growth hormones, and dietary nutrients (5). Immune activation is energetically costly and can drive physiological changes associated with undernutrition as well as being caused by them (5). For example, persistent low level inflammation is associated with reduced adiposity and lean mass deposition among Gambian adolescents (21). Micronutrient homeostasis can also be influenced by infection; for example, acute phase proteins inhibit intestinal iron absorption and promote sequestration of circulating iron in macrophages to reduce availability for extracellular pathogens, thereby causing iron-deficiency anemia and altered macrophage function (22). The combined effect of undernutrition on immune cell function and its impact on infectious susceptibility are not well-characterized for children in LMIC. This reflects both a lack of studies and the difficulty of teasing out causal pathways in the context of multiple concurrent immune challenges. Studies among undernourished children to-date have tended to focus on the immune system as a biomarker of undernutrition rather than as an inherent part of the undernourished state (5).

In the past decade, substantial advances in experimental approaches to study the microbiome have been used to demonstrate a causal association between dysbiosis in the gut and malnutrition [extensively reviewed elsewhere; e.g., (13, 23)]. However, despite the reciprocal relationship between the immune system and the microbiome, few translational studies of the microbiome of undernourished children in LMIC have assessed innate immune cell function. Later in this review, we will discuss how existing malnutrition models could be improved to understand microbiome-immune interactions.

An emerging paradigm that may be critical to our understanding of innate immune cell function in undernutrition is innate immunological memory, which arises independently of T- and B-cells, including a process termed “trained immunity.” Innate immune cells can be “trained” by environmental exposures, improving their secondary response to infection (24). For example, BCG vaccination is associated with heterologous protection against all-cause mortality, including non-mycobacterial infections (25). Human monocytes can also be “trained” by micronutrients *in vitro*. Addition of Vitamin A to monocyte cultures treated with BCG led to an inhibitory histone methylation mark (H3K9me3) that suppressed monocyte cytokine responses upon re-stimulation relative to monocytes treated with BCG alone (26). Recent studies of allergic airway inflammation (27), vaginal candidiasis (28), and cutaneous wounding in mice (29) also provide a proof-of-principle that epithelial cell responses can be “trained.” “Training” can also be deleterious, since innate immune cell adaptation to one context may render cells less able to defend against distinct challenges. One example is the “immunoparalysis” that can arise after sepsis, whereby innate immune cells down-regulate pathogen recognition receptors, co-receptors and pro-inflammatory cytokines to prevent immunopathology but, as a result, are hyporesponsive to new infections (30). Maintaining metabolic plasticity is necessary for immune cells to generate anti-pathogen responses when challenged and this appears to be compromised in immunoparalysed cells. For example, monocytes from healthy adults injected with endotoxin have a less pronounced metabolic response to re-stimulation with endotoxin and an associated reduction in pathogen killing *in vitro* 7 hours post-injection relative to monocytes isolated from donors without pre-exposure to endotoxin (31). Trained immunity may be one mechanism by which innate immune cells adapt to the simultaneous derangement of microbial and nutrient exposures during undernutrition. Children with SAM share many of the clinical features of sepsis, which is a common clinical complication of SAM and a recognized stimulus for monocyte training (31–33). During sepsis, epigenetic modifications in monocytes down-regulate pro-inflammatory cytokine production but upregulate alternative functional genes, including antimicrobial peptides (AMP) (32, 33). It is unclear whether adaptation of monocytes (or other innate immune cell types) is affected by SAM. Stunting has been shown to alter histone methylation patterns in blood cells from Bangladeshi children during the first 2 years of life, including H3K4me3 marks at metabolic and immune gene sites (34). Of the pathways mapped to the H3K4me3-marked genes that were also positively associated with linear growth, the immune system was the top hit and gene expression analysis identified enrichment of innate responses including IL-6 and Toll-like receptor (TLR) signaling in stunted children (34). The implications of these changes for immune cell function and clinical outcomes are unknown, but warrant further study.

### Biomarkers of an Undernourished Innate Immune System

Most immunology studies to-date have focused on soluble inflammatory mediators. Undernourished children in LMIC have higher levels of inflammatory biomarkers than adequately nourished children (16, 35) or after nutritional rehabilitation for SAM (10, 36). Some studies have reported negative associations between a range of pro-inflammatory mediators, including C-reactive protein (CRP; a liver acute phase protein), S100A12 (a gene associated with pro-inflammatory leukocyte activation), soluble CD14 (a biomarker of monocyte activation), calprotectin and myeloperoxidase (anti-microbial peptides enriched in neutrophils), and neopterin (a protein released by activated macrophages), and height-for-age Z scores (37–40), weight-for-age Z scores (38, 39), and MUAC (41). A causal role for inflammation in stunting and wasting is thought to result from suppression of growth hormone signaling [e.g., insulin-like growth factor (IGF) 1 (16, 17) and IGF binding protein (IGFBP) 3 (16)] by inflammatory mediators, and/or the increased resting energy expenditure incurred by chronic immune cell activation and inflammatory mediator synthesis. However, immune biomarkers are non-specific and cannot provide information on the cellular source, anatomical site or stimulus for their production. Furthermore, existing levels of inflammatory mediators do not predict the capacity of innate immune cells to respond to subsequent infectious challenge.

Even among children who are not clinically undernourished, the gut is a critical site of inflammation in the context of high pathogen prevalence and marginal diets. In these settings, an almost ubiquitous sub-clinical condition termed environmental enteric dysfunction (EED) is present and associated with colonization with pathogenic microorganisms, dysbiosis, innate leukocyte recruitment, and reduced gut functional capacity (12). Not all children with EED are malnourished, however reduced gut surface area and function due to EED may act as a barrier to healthy nutrition among children with an adequate nutrient intake and during therapeutic feeding of children who are already stunted and/or wasted (12, 42). A systematic review of the hypothesized pathways linking EED and stunting found evidence that intestinal inflammation is associated with systemic inflammation and reduced linear growth (12), but the mechanisms underlying this association are poorly characterized. The most frequently measured biomarkers of intestinal inflammation are proteins associated with accumulation of innate immune cells in the gut mucosa: myeloperoxidase, calprotectin, and neopterin (12). In a birth cohort study conducted in 8 LMIC (Malnutrition and the Consequences for Child Health and Development; MAL-ED), the relationship between inflammatory biomarkers and child growth was driven by sub-clinical enteropathogen carriage, with systemic inflammation most strongly associated with reduced linear growth and intestinal inflammation more closely associated with reduced ponderal growth (39).The function of innate immune cells was not characterized in MAL-ED and it therefore remains unclear whether inflammation is a by-product of heightened infection or results from cellular dysfunction.

Studies among children hospitalized with SAM suggest that systemic and intestinal inflammation independently contribute to mortality (8, 43, 44). High levels of circulating CRP at hospital admission were predictive of inpatient mortality in children admitted with SAM to hospitals in Uganda (44) and Niger (43). Among 79 children aged 6–59 months with complicated SAM in Malawi, children who died during hospitalization had higher levels of fecal calprotectin and plasma inflammatory cytokines (Growth colony stimulating factor (G-CSF), IL-6, TNFα, IL-1Rα, IL-13, TNFβ, and IL-2) (8). Plasma levels of the short-chain fatty acids butyrate and propionate, which regulate inflammatory signaling, were also lower among those who died (8). Kenyan children with SAM who died within 60 days of hospital discharge also had higher levels of TNFα, G-CSF, IL-8, IL-15, and interferon gamma-induced protein 10 (IP-10) than those who survived without readmission to hospital for up to 1 year (9). In the same study the plasma proteome profile of children who died indicated elevated innate immune cell anti-pathogen responses and acute phase proteins (9). These studies make clear that both short- and longer-term mortality in complicated SAM has an inflammatory component. However, the prevalence of infections among children enrolled in these studies was high and many of the inflammatory biomarkers of mortality are also increased during clinical and sub-clinical infections, even in adequately-nourished children. Whilst elevated inflammation during childhood undernutrition in LMIC may simply indicate patent infection, it is also possible that undernutrition-driven immune dysfunction increases infectious susceptibility and/or worsens clinical outcomes among infected children. For example, Versloot and colleagues found that pathogen carriage among children with complicated SAM did not predict inpatient mortality and, whilst fecal calprotectin levels correlated with pathogen carriage, systemic CRP levels did not (10). The relative importance of immune function and infection on the clinical outcomes of undernutrition remains an open question, which cannot be resolved using non-specific inflammatory biomarkers alone. The persistent burden of infectious mortality after nutritional rehabilitation from SAM, indicates that the capacity of a child's immune system to respond to new infections should be assessed alongside their existing levels of inflammation.

### Evidence for Innate Immune Cell Dysfunction in Undernutrition

Inclusion of functional analysis of immune cells in cohort studies of children in LMIC is uncommon. Of the studies conducted, many demonstrate innate and adaptive immune cell dysfunction during undernutrition [summarized in a systematic review by Rytter et al. (45)]. However, most are limited by their small sample size, varying definitions of malnutrition, lack of appropriate controls, and cross-sectional designs; the majority of studies pre-date current immunological techniques to resolve cell-type specific responses. Furthermore, most existing studies of innate immune cell function in undernutrition have not evaluated the clinical implications of the cellular defects identified. We are left with the pertinent question: do defects identified in innate immune cell function leave undernourished children more vulnerable to infectious morbidity and mortality, or are they merely a consequence of defects in other physiological pathways (5, 46)?

The strongest evidence for innate immune cell dysfunction comes from hospital-based cohort studies of complicated SAM (45). However, disentangling the effects of infection and undernutrition is often not possible in complicated SAM since the majority of children also have symptomatic infections (6–8, 10). Longitudinal studies provide an opportunity to compare immune cell function between children at admission, when infections are present, and during recovery when children are still wasted but infections have been treated with broad-spectrum antibiotics. However, characterizing immunological recovery is challenging since “healthy” immune function is poorly defined and sub-clinical pathogen carriage (47–49) and enteropathy (50) are common even among adequately-nourished children in LMIC. Some studies have enrolled adequately-nourished children from high-income countries (HIC) as controls, but their utility in determining immunological thresholds for children in LMIC is limited by their vastly different developmental and environmental exposure histories. For example, the innate immune system has developed under intense selection pressure from infectious diseases and there are clear regional distinctions between innate immune cell ontogeny and function that are dependent on local pathogen exposure patterns (51–53). Of particular relevance to evaluating innate immune cell function in African cohorts, selection against erythrocyte expression of Duffy antigen (also called atypical chemokine receptor 1) by endemic exposure to *Plasmodium* parasites has shaped a distinct myelopoietic niche in the bone marrow resulting in a characteristic neutropenia among the majority of people of African ancestry (53). A further socio-economic challenge to longitudinal studies of immune function in children with complicated SAM is that they tend to come from marginalized families with high mobility, meaning that follow-up rates are often low (7, 54, 55).

HIV overlaps with malnutrition, and shares many common underlying pathogenic pathways (e.g., chronic inflammation, immune activation, and enteropathy). The interaction between HIV, inflammation and undernutrition was demonstrated in a recent study of children starting antiretroviral therapy in Uganda and Zimbabwe (56). A proportion of children with advanced baseline immunosuppression and low WAZ were hospitalized for complicated SAM several weeks into ART, and this clinical deterioration was independently associated with concentrations of several inflammatory biomarkers (e.g., IL-6 and TNFα) (56). HIV therefore impacts both immune function and nutritional status (55), meaning that insights into immune function from studies of undernourished children conducted prior to routine HIV testing [e.g., (57–65)] or including HIV-positive children [e.g., (66, 67)] cannot be generalized to an effect of undernutrition *per se*. Children with SAM and HIV have 4-fold higher mortality than HIV-uninfected children with SAM and three times higher mortality than would be expected from their anthropometry alone (68). Carefully designed studies that stratify undernourished and adequately-nourished children by HIV status are therefore required to better understand the independent effects of both conditions on innate immune function.

**Figure 1:**
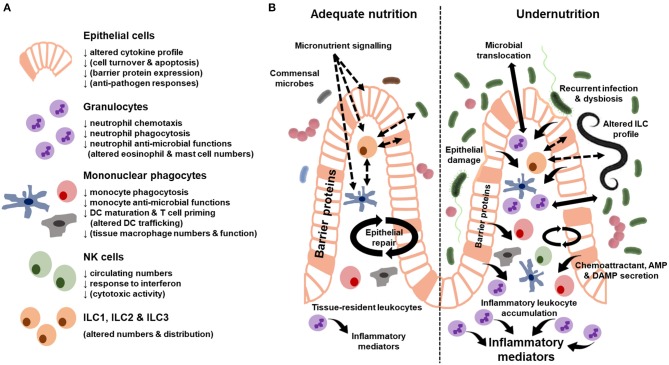
Summary of innate immune cell dysfunction during undernutrition. **(A)** Cellular functions where there is evidence of dysfunction from human cohort studies and animal models of undernutrition. Functions in brackets only have evidence from animal models. **(B)** Innate immune characteristics of the adequately-nourished (left) vs. undernourished (right) gut. Solid arrows indicate secreted proteins and cell behavior. Dashed arrows indicate signaling pathways. Differences in the size of arrows and text indicate quantitative differences in the response between the adequately-nourished and undernourished state. AMP, antimicrobial peptides; DAMP, damage-associated molecular patterns.

No studies to our knowledge have assessed innate immune cell function in stunting in LMIC beyond soluble inflammatory biomarkers (discussed above). There has also been limited assessment of innate immune cell function in undernourished tissues, with an understandable majority of studies focusing on more readily accessible innate immune cells in peripheral blood. Despite the limitations of existing studies, a number demonstrate innate immune cell dysfunction in undernutrition (summarized in Figure 1A) and there is emerging evidence that this exacerbates the undernourished state (5).

#### Epithelial Cells

Epithelial cells have a critical role in innate barrier defense. They sense changes in the diet, microbiome and enteropathogens, and respond to these signals by secreting AMP and transmitting signals that recruit leukocytes from the blood and maintain local niches for gut-resident immune cells. In the healthy gut, the epithelium can compartmentalize apical (from the gut lumen) and basal (from the lamina propria) signaling via polarized expression of receptors and tight regulation of membrane permeability. Immunohistochemical analysis of intestinal biopsy specimens indicates that this regulation by epithelial cells is disrupted in children with SAM (summarized in Figure 1B). Mechanical damage to the epithelial barrier ranges from localized abrasions and loss of tight junction proteins between epithelial cells to widespread reductions in epithelial surface area due to villous atrophy (69). There is an associated increase in leukocyte infiltration into the lamina propria (50, 69), which may be driven by a number of signals, including damage-associated molecular patterns (DAMPs) and chemoattractants released by activated epithelial cells and tissue-resident immune cells. One consequence of these epithelial changes is increased gut permeability (12, 69, 70), which can be measured indirectly by increased urinary excretion of compounds that are usually not absorbed (e.g., lactulose) and/or increased blood levels of bacterial antigens that would usually be retained on the apical side of the epithelium in the gut lumen (microbial translocation) (12). Intestinal permeability can be increased both directly via gastrointestinal infections, as seen among undernourished children with sub-clinical enteropathogens or diarrhea (12, 69), and indirectly via pro-inflammatory signals resulting from infections outside the gut (71). Recurrent clinical and sub-clinical gastrointestinal infections and ongoing systemic inflammation may therefore worsen gut barrier function and nutrient absorptive capacity during undernutrition.

Few human studies have directly assessed epithelial cell function in undernutrition. However, one study identified greater immunohistochemical staining for the immune regulating cytokine TGFβ in epithelial cells (as well as T cells) in gut biopsy specimens from severely undernourished Gambian children compared to adequately-nourished controls from the same community (72). Epithelial cell responses have been demonstrated in more detail in experimental animal models of undernutrition (73–76), though few models consistently recapitulate the villous atrophy and leukocyte infiltration observed in intestinal biopsies from human undernutrition (69). The sub-clinical features of children in LMIC, such as EED and milder growth defects, are particularly challenging to model in the laboratory without overt pathogen challenge (76). However, in a model of colitis resulting from food poisoning, delivery of recurrent non-lethal doses of *Salmonella enterica* serovar Typhimurium to adequately-nourished mice progressively depleted gut epithelial cell surface expression and secretion of intestinal alkaline phosphatase (77)—a mechanism which detoxifies bacterial endotoxin and limits pro-inflammatory signaling via the endotoxin receptor TLR4. Recurrent infections occurring in children with EED may also deplete intestinal epithelial cell anti-inflammatory mechanisms, and thus compromise gut functional capacity and/or promote training of local leukocytes. Undernourished animals also display an altered epithelial immune response to infections. Growth defects in undernourished weanling mice are accompanied by decreased epithelial turnover and increased epithelial cell apoptosis in the jejunum (74). Murine gut epithelial cells also fail to proliferate or activate caspase-3-driven apoptosis of infected cells to the degree observed in adequately-nourished animals, resulting in higher parasite numbers in the ileum during *Cryptosporidium* infection (73). Intestinal epithelial cells isolated from gnotobiotic pigs with protein energy malnutrition (PEM) and colonized with the fecal microbiota of healthy human infants have reduced mRNA expression of MUC2 and Villin, which encode critical barrier proteins found in enteroendocrine cells and brush border enterocytes, respectively (75). After exposure of the piglets to rotavirus, epithelial cells from undernourished piglets expressed lower MUC2, Villin, chromogranin A, proliferating cell nuclear antigen and SRY-Box 9 than adequately-nourished rotavirus-infected controls (75). Defects in the intestinal barrier were associated with greater translocation of *Clostridium perfringens* type A and *Escherichia coli* to the liver, kidney, lung, and peritoneal cavity and delayed clearance of rotavirus infection (75). Thus, barrier defects in undernutrition are exacerbated by defects in epithelial cell function, which contribute to increased duration and severity of infection.

Disrupted epithelial cell responses have also been observed in enteropathy associated with other health conditions. For example, diabetic enteropathy in adults causes defects in colonic epithelial stem cell mobilization due to reduced levels of circulating IGF-1 and IGFBP3 (78), both of which are also low in children with stunting (16, 37). Furthermore, short periods of dietary restriction (fasting for 32 and 72 h), alter epithelial AMP production, reducing Reg3β, Reg3γ, and Saa1 mRNA, but retaining α-defensin (79). It is therefore plausible that epithelial cells in the gut, and potentially at other barrier sites, functionally adapt to malnutrition enteropathy in a way that changes their secondary responsiveness to damage and infection. It is not known whether such compensatory epithelial responses can be maintained with more severe and/or longer-term nutrient deficiency and concurrent physiological defects during childhood undernutrition in LMIC. However, correcting epithelial cell function may be necessary to restore chronic barrier defects in undernutrition.

#### Granulocytes

Granulocytes are the most studied innate immune cell type in childhood undernutrition in LMIC (45). 19 of the 29 studies of innate immune cell types conducted between 1970 and 2014 focused on granulocytes (45); of these, 8 focused on neutrophils and none isolated basophil, eosinophil or mast cell functions (45). Neutrophils respond rapidly to stimuli by accumulating at the site of damage or infection and releasing pre-formed granules containing AMP and pro-inflammatory mediators. Although principally known for their pro-inflammatory functions, neutrophils also play a role in tissue homeostasis and resolution of inflammation (80), which has not been investigated in human undernutrition. The few undernutrition studies that have obtained gut biopsy specimens demonstrate that granulocyte numbers are markedly expanded in mucosal tissue, but this is most likely due to unresolved infection and there is limited evidence for abnormal numbers of circulating blood granulocytes caused by undernutrition *per se* [reviewed by Rytter et al. (45)].

One of the main functions of neutrophils is to migrate from the blood into tissues in response to chemokine gradients and accumulate where chemokine concentrations are highest. Studies conducted in the 70s were the first to show that blood neutrophils isolated from undernourished children have reduced chemotaxis (58, 59), but the cause of this impairment is disputed. These studies were also subject to methodological limitations due to the propensity for neutrophils to become rapidly activated during laboratory handling, particularly using older isolation and culture methods. Chemotactic deficits have also been observed in total granulocyte populations during undernutrition (63, 81, 82), with similar methodological limitations. Reduced chemotaxis of granulocytes from HIV-negative Mexican children with SAM was restored after 4 weeks of nutritional therapy relative to hospital admission (82), although there was no healthy control group included in the study to determine whether responses fully normalized. Results from a small study (*n* < 10 per group) of adults with visceral leishmaniasis also demonstrated that wasting reduced biomarkers of granulocyte chemotactic capacity, including lower surface expression of selectins and integrins (CD62L and CD11b) and lower granulocyte chemoattractant secretion (IL-8 and MIP1α) (81). Whilst these studies in separate cohorts broadly agree that granulocytes migrate less efficiently during undernutrition, early observations require further validation using more reliable assays, larger cohorts and systematic separation of the effects of infection vs. nutritional status.

Upon arrival at the site of tissue damage or infection, granulocytes engulf pathogens via phagocytosis and secrete intracellular and extracellular AMP and ROS to enhance clearance of infection. Reported effects of undernutrition on the phagocytic ability of granulocytes are inconsistent; some studies show that phagocytosis is unaffected (57, 58, 63, 83), whilst others observed reduced phagocytosis (82, 84). Inconsistencies between studies may reflect differences in assay choice as well as the distinct geographical, age and disease contexts in which the studies were performed. There is more consistent evidence that granulocyte anti-microbial functions are reduced by undernutrition, with evidence for lower *in vitro* production of ROS (61, 63, 81, 83, 84) and impaired *in vitro* killing of Candida (63) and bacteria (57, 84, 85). Among children hospitalized with kwashiorkor in Cote d'Ivoire, bactericidal activity of peripheral blood phagocytic cells (mononuclear cells and granulocytes were not distinguished) against *E. coli* and *S. aureus* during the first 30 min of co-culture was normal when compared to healthy controls from the same community, but reduced after 60 min (57). Assessment of ROS production by granulocytes from undernourished children using the nitroblue tetrazolium test has yielded inconsistent results across studies (45). Reports of impaired metabolic (58, 63) and antimicrobial responses (61, 63) during phagocytosis suggest that granulocytes may have an impaired capacity for intracellular killing even if phagocytic uptake is preserved. Several studies which simultaneously assessed multiple granulocyte functions indicate that some are impaired whilst others are intact (57, 58, 63), indicating that undernutrition may not uniformly reduce granulocyte anti-microbial functions.

Basophils and eosinophils have distinct roles from neutrophils and are critical for defenses against parasitic worms (helminths) and during allergic responses (86). Helminth infections are highly prevalent in LMIC and can directly drive nutritional deficits through feeding on gut luminal contents, blood and circulating nutrients (87). These parasites may also exacerbate undernutrition indirectly through mechanical damage to the gastrointestinal tract through feeding (e.g., hookworm), egg deposition in tissues (e.g., *Schistosoma mansoni*) or luminal obstruction in high-intensity infections (e.g., *Ascaris*) (87). There is some evidence that undernutrition also increases the risk of helminth infections (88), but no studies have investigated the impact of childhood undernutrition on the anti-helminth responses of granulocytes. In rats, PEM impaired systemic and mucosal immune responses to *Trichinella spiralis*, including reduced circulating eosinophils (89). However, vitamin D deficiency increased spontaneous activation of eosinophils in the lamina propria (90). A study among children in the USA found that allergic responses during atopic dermatitis, including elevated eosinophil and basophil counts, were associated with low bone mineral density, as was a body-mass-index <5th centile (i.e., underweight) (91). These studies indicate that basophil and eosinophil dysfunction warrant further study in human undernutrition, where their dysfunction may have knock-on effects on associated risk factors including helminth infection, gut function and bone density.

Mast cells also play a role in anti-helminth responses and allergy, however their localization in connective tissue has been a barrier to studying their function in undernourished cohorts. Animal models suggest that mast cell accumulation may be deregulated by macro- and micronutrient deficiencies in ways that reduce anti-helminth responses and increase inflammatory disease. In PEM rats infected with *T. spiralis*, the impaired systemic and mucosal immune response to infection was also associated with fewer mast cells in airway and intestinal mucosae (89). During magnesium deficiency, which exacerbates liver fibrosis in a rat model, depletion of mast cell numbers in the ileum, kidney, and bone marrow occurred alongside increased mast cell accumulation and functional gene expression (α- and β-chain high-affinity IgE receptors, mast cell protease 1 and 2) in the liver (92).

#### Monocytes, Macrophages and Dendritic Cells

The mononuclear phagocyte system, made up of monocytes, macrophages and dendritic cells (DC), bridges the gap between the innate and adaptive immune system. These “professional” antigen-presenting cells are critical for detection, uptake and processing of pathogen antigens, which they use to activate antigen-specific T cells in secondary lymphoid organs. Maturation of mononuclear phagocyte antigen-presenting capacity can be monitored by upregulation of membrane expression of major histocompatibility complex (MHC; e.g., HLA-DR) and co-stimulatory molecules (e.g., CD86, CD80) in response to pathogen antigens.

Monocytes are innate effectors and precursors to some types of macrophages and DC. Monocyte maturation is accelerated in the context of sepsis (32, 93) and experimental exposure of healthy adult volunteers to intravenous endotoxin (94, 95). Within circulating monocyte populations, so-called classical monocytes (CD14^hi^CD16-) mature into intermediate (CD14^hi^CD16+) and then non-classical sub-types (CD14^lo^CD16+), reflecting a shift in their function. A variety of inflammatory diseases are associated with expansion of the intermediate monocyte population (95, 96). Monocyte ontogeny and sub-types have not been characterized in human undernutrition. Furthermore, initial steps in pathogen recognition by mononuclear phagocytes (or any immune cell type) and their pathogen recognition receptor repertoire during undernutrition are unknown (5, 45). Alternative monocyte functions are better characterized. For example, when compared to healthy controls, a lower proportion of monocytes from HIV-negative Brazilian children with SAM phagocytosed *Saccharomyces cerevisiae* and they also produced lower amounts of nitric oxide and superoxides (97). The defect in ROS production was partially restored by nutritional rehabilitation, but remained lower than controls throughout the study (97). Reduced monocyte phagocytosis has also been observed in Egyptian infants with undernutrition (98). Complement components and the opsonising ability of serum are reduced in undernourished children (60, 98), which may contribute to impairments in phagocytic activity. Such defects in the interactions between monocytes and pathogens might be expected to impair clearance of infection by monocytes themselves and compromise T cell priming due to delayed antigen processing.

A single study has investigated DC phenotype and function during childhood undernutrition in LMIC. Children admitted to hospital with SAM in Zambia had low numbers of myeloid DC at admission and this population of cells expanded over the course of nutritional rehabilitation (66). Children with low baseline DC numbers also had defects in monocyte function (lower HLA-DR expression and endotoxin-induced TNFα production *in vitro*) and an increased risk of mortality compared to children with normal DC numbers (66). The antigen-presenting capacity of DC from most children matured normally in response to endotoxin stimulation *in vitro*; however, DC from a sub-set with endotoxemia (~17%) did not upregulate HLA-DR (66). A role for endotoxemia in driving DC dysfunction during undernutrition was supported by assays showing that the T cell priming capacity of DCs was inversely associated with circulating endotoxin levels (66). Defects in DC function during undernutrition have also been demonstrated in murine models. For example, PEM reduced splenic DC numbers and impaired their ability to prime virus-specific T cells in response to the hepatitis B vaccine (99). Provision of the DC growth factor Flt3 ligand restored delayed-type hypersensitivity responses in a murine model of weanling undernutrition (100), an indicator that impaired responses were partially due to defective DC expansion. In undernourished mice infected with *Leishmania donovani*, fewer monocyte-derived DC, but not migratory DC, accumulated in the skin-draining lymph nodes and there was lower lymph node expression of some DC chemokines and DC expression of the chemokine receptor CCR2 (101). In contrast, DC and macrophages that migrated to the lymph nodes via alternative chemokine signals were increased (101), suggesting that the relative roles of DC sub-types may change during undernutrition to alter the anti-pathogen response and/or compensate for defective DC migration. In piglets fed a protein-deficient diet, the frequency of plasmacytoid DC and CD103+ DC numbers were lower in the duodenum and ileum but normal in the spleen (75), which also indicates that DC trafficking may differ between physiological sites. The effect of undernutrition on mononuclear phagocyte recruitment to barrier sites in humans is currently unknown.

Although some macrophages derive from circulating monocytes, many tissue-resident macrophages are seeded during embryonic development (102). The basic biology of these distinct macrophage populations is an emerging field that has not been translated to cohort studies of childhood undernutrition in LMIC. However, several experimental animal models demonstrate impairments in tissue macrophage function during PEM, including reduced numbers in the peritoneal cavity, lymph nodes and alveoli, and reduced phagocytosis and endotoxin-responses similar to those seen in human blood monocytes [reviewed by Ibrahim et al. (103)]. PEM in pregnant rats also led to differences in the alveolar macrophage responses of their offspring; macrophages isolated from pups of malnourished dams had lower expression of TLR9 and higher expression of NFκB (a transcription factor down-stream of TLR mediating pro-inflammatory cytokine signaling) when cultured with *Staphylococcus aureus* than those isolated from pups of protein-sufficient dams (104).

In addition to their anti-pathogen responses, mononuclear phagocytes directly sense micronutrients via surface receptors. Micronutrient deficiencies drive changes in monocyte, macrophage and DC sub-sets, their distribution and function [summarized elsewhere (5)]. For example, iron transport in macrophages influences their antibacterial responses (22). Vitamin A treatment during *in vitro* monocyte activation leads to an incremental reduction in pro-inflammatory cytokine production mediated via inhibitory histone modifications at cytokine gene promoter sites (26). In blood samples from healthy adult volunteers, *in vitro* vitamin D supplementation had distinct effects on pro-inflammatory endotoxin signaling in monocytes, monocyte-derived macrophages and monocyte-derived DC, with increased phosphorylation of the pro-inflammatory transcription factor NFκB in monocytes, no effect on macrophages and inhibition in DC (105). Micronutrient deficiencies are highly prevalent in LMIC across the spectrum of undernutrition (19, 106); their impact on innate immune cell micronutrient sensing in this context has not been characterized.

#### Natural Killer Cells

Natural Killer (NK) cells are innate lymphocytes that can rapidly respond to virus-infected and cancerous cells independently of antigen presentation via MHC or antibodies. Studies of undernourished children in sub-Saharan Africa have found lower NK cell numbers than in adequately-nourished children, despite similar NK cell proportions [summarized by Rytter et al. (45)]. In a small number of undernourished Mexican children with infections, NK cell numbers were also lower than adequately-nourished children with or without infections; numbers were not restored by nutritional rehabilitation (107). Defects in NK cell numbers in a Nigerian cohort coincided with low serum interferon levels and hyporesponsiveness to interferon stimulation *in vitro* (108); both defects normalized after nutritional recovery (109). Interferon production and NK cell responses are a hallmark of innate anti-viral responses. NK-derived IFNγ also promotes Th1 polarization of CD4+ T cells, enhancing anti-bacterial and anti-viral adaptive immunity. A genome-wide association study of children in Malawi and Kenya found that STAT4 polymorphisms were associated with an increased risk of non-typhoidal salmonella, which was related to low IFNγ production by NK cells and decreased NK cell responsiveness to IFNγ during infection (110). The association between NK cell function and the risk of salmonellosis has not been investigated in undernutrition, however non-typhoidal salmonella infection is common in children with complicated SAM at hospital admission (6, 8) and during suspected sepsis (7), suggesting that NK cell dysfunction may contribute to their risk of salmonellosis.

Although data on NK cell function in human undernutrition are limited, animal models have again been useful to provide a proof-of-principle that NK cell dysfunction may contribute to adverse outcomes. In rotavirus-infected piglets, NK cell numbers were lower in the spleen, duodenum and ileum of protein-deficient animals compared to protein-sufficient animals and NK cells from protein-deficient animals had a reduced capacity to kill a cancer cell line *in vitro*, even when NK-to-tumor cell ratios were high (75). Defects in NK cells and other cell types (discussed above) in the protein-deficient piglets were associated with higher peak viraemia and longer periods of diarrhea during rotavirus infection, which coincided with greater mortality, weight loss and intestinal barrier dysfunction (75).

Whilst calorie restriction without undernutrition is thought to improve longevity in high-income settings (111), animal studies suggest that calorie-restricted NK cells are less effective in the context of infection. Calorie restriction led to elevated *in vitro* production of TNFα and GM-CSF but reduced IFNγ by NK cells activated with cytokines (IL-2 plus IL-12) or antibody ligation of NK1.1 surface antigens relative to NK cells isolated from *ad libitum*-fed controls (112). A murine model of influenza demonstrated that mortality was greater in calorie-restricted vs. *ad libitum*-fed animals due to NK cell defects (113), which were rapidly restored by re-feeding (114). It is plausible that NK cell dysfunction resulting from chronic undernutrition or more severe nutritional deficits, such as during SAM, may also lead to impaired protection against common pathogens via similar mechanisms. Lower pathogen exposure, milder calorie restrictions and implementation of dietary changes during adulthood rather than in early life may explain the health benefits of calorie restriction seen in studies conducted in HIC (111).

#### Innate Lymphoid Cells

Innate lymphoid cells (ILC) share some of the functions of adaptive T cells, but respond to challenges with the rapid kinetics of innate immune cells [reviewed by Eberl et al. (115)]. NK cells are considered a type of ILC with analogy to CD8+ T cells (see above), whilst ILC1, 2 and 3 are more like CD4+ T cells of the Th1, Th2, and Th17 phenotype, respectively (115). ILC play a critical role in barrier defense, promoting epithelial repair and local leukocyte functions. ILC2 function is dependent on lipid metabolites, whilst ILC3 function is more closely related to dietary nutrient sensing, particularly aryl hydrocarbons and vitamin A [eviewed by Wilhelm et al. (116)]. The differences in the cellular metabolism of ILC sub-sets may be particularly relevant to undernutrition, where nutrient metabolites are limited by a marginal diet, lower gut absorptive capacity and disrupted host and microbiome nutrient metabolism. Furthermore, since monocyte metabolic plasticity can be reduced by experimental endotoxin exposure (31), it is plausible that chronic endotoxin exposure in the context of undernutrition may also compromise ILC responses. Murine models demonstrate reciprocal adaptation of gut ILC sub-sets to micronutrient deficiency, whereby vitamin A deficiency depletes ILC3 in favor of ILC2 (117, 118), and aryl hydrocarbon receptor signaling promotes ILC3 numbers over ILC2 (119). During concurrent helminth infection and vitamin A deficiency, ILC2 increased their uptake of extracellular fatty acids and prioritized IL-13 and mucus production to maintain anti-helminth responses (120). As relatively newly-defined cell types, our understanding of ILC function is developing rapidly but is yet to be translated into human studies of undernutrition. Future studies will be critical to understand how ILC sub-types adapt to simultaneous micro- and macro-nutrient deficiencies and chronic enteropathy. For example, optimal release of short-chain fatty acids from fermentable carbohydrates relies on dietary fiber intake and microbiome composition (42, 121).Furthermore, the profound metabolic dysregulation in children with complicated SAM may have implications for ILC-mediated barrier functions (122).

## Improving Assessment of Immune Function in Undernutrition

### Cohort Studies in LMIC

In this review we have outlined knowledge gaps in the function of all innate immune cell types during childhood undernutrition. A concerted effort is needed to translate immunological paradigms from animal models into human cohort studies. Furthermore, whilst assays of soluble immune biomarkers, proteome, transcriptome, epigenome, and metabolome profiling in peripheral samples provide insights into immune and immune-metabolic derangement, direct measurements of immune function will rely on cell- and tissue-based assays. In the long-term, developing high-quality assays of innate immune cell function for well-powered population studies in LMIC will rely on leveraging existing laboratory capacity for immunology research and developing capacity where it is not currently available. Such efforts would be accelerated by adaptation of existing technologies for assessing immune cell function in high-income settings to affordable standardized field-deployable formats. Cohort studies of innate immune function should be: (i) powered adequately to detect differences in highly heterogeneous immune responses; (ii) longitudinal in design to distinguish short-term fluctuations from persistent functional defects; and, (iii) ideally nested within studies that also characterize concurrent immunological stimuli (e.g., pathogen carriage, micronutrient deficiencies, metabolic and microbiome characteristics, enteropathy), which are necessary to accurately interpret immune cell behavior. To identify localized changes in innate immune cells and learn more about the function of tissue-resident cells during undernutrition, sampling from a more diverse range of anatomical sites will be necessary. For example, despite respiratory tract infections being a frequent cause of mortality among undernourished children (1), we know almost nothing about the airway immune response. Very few studies have assessed inflammatory biomarkers or immune cells in bronchiolar lavage fluid, saliva, naso-gastric aspirates, or tissue biopsies. Even relatively straightforward assays of cell function using a wider range of sample types would provide important advances to the field of nutritional immunology.

Beyond understanding the basic immunology of childhood undernutrition, developing novel therapies targeting innate immune dysfunction will require cohort studies to assess whether innate immune cell function is associated with clinical outcomes, and identify the pathways that lead to dysfunction. Identifying immune biomarkers of prognostic value for stunting, wasting and infectious mortality has proven challenging to-date due to the multiple mediators that are simultaneously altered (8, 10, 12). Thus, where possible, future studies should make use of multiparameter assays of immune cell function and consider the value of using composite scores from multiple biomarkers for triaging patients (5).

### Experimental Models

Most of the immunological paradigms for innate immune defects shaping long-term infectious susceptibility come from experimental animal models. Animal models provide the opportunity to precisely and reproducibly define nutrient intake, microbial exposures and environmental conditions relevant to undernutrition. They permit access to cells and tissues from anatomical sites that are usually inaccessible and genetic/pharmacologic manipulation that are not ethical to undertake in humans. Judicious application of refined models has substantially advanced our understanding of complex diseases that also depend on multiple concurrent environmental exposures, including inflammatory bowel disease (123). Likewise, our basic understanding of how different nutrients influence immune cellular function and *vice versa* has been bolstered by studies of nutrient deprivation in animals (121). Few of these insights have been translated into studies of undernutrition in LMIC. To better leverage animal models for translational research in childhood undernutrition, they must be carefully designed to better recapitulate “real-life” settings in LMIC. In particular, model diets, microbial, and environmental exposures could be readily adapted for this purpose.

#### Model Diets

Most animal models of undernutrition use dietary restrictions aimed to generate overt deficiency phenotypes, and measures used to induce nutritional deficiency are sometimes extreme. For example, vitamin A-deficient rodents are not just fed a diet completely lacking in vitamin A from weaning, but are often born to dams established on a deficient diet from mid-gestation and throughout the post-natal period (117). Micronutrient signaling pathways can also be completely ablated in genetic knock-outs or conditional genetic knock-outs (117). Acutely malnourished children are generally deficient in multiple nutrients concurrently (19, 106), but the pattern and extent of deficiencies is extremely heterogeneous (both between individuals in the same setting, but especially between different settings and regions) and abnormalities in nutrient trafficking mean some body compartments may even experience nutrient excess. For example, despite whole-body iron deficiency, free iron may be high in plasma due to protein deficiency limiting production of the iron-transporter protein transferrin (124, 125) or actively sequestered in macrophages during infection to restrict iron availability for pathogens (22). Ideally, diets used for animal models of undernutrition should be based on either: (i) nutrient intake profiles of undernourished children in a defined setting, or (ii) levels of existing nutrients, ideally taking into account the circulating nutrient pool as well as any tissue stores. Both approaches have limitations. While a few studies on undernutrition in rodents have attempted to recreate subsistence diets and ready-to-use therapeutic food interventions relevant to LMIC [e.g., (126–128)], detailed analyses of the recent nutrient intake of children with acute undernutrition are surprisingly rare in the literature, labor intensive, subject to recall bias, and difficult to undertake sensitively without reinforcing the stigma for caregivers of undernourished children (129). There is a need for better cohort studies of the real rather than perceived nutrient gaps in undernourished children to inform model diets, particularly multi-pass methods that comprehensively record 24-h dietary intakes on several occasions and identify nutrient gaps by comparing actual intakes with recommended intakes for age. Furthermore, chronic inflammation and recurrent infections alongside dysregulated nutrient uptake, transport and storage among undernourished children in LMIC may incur increased basal nutrient requirements compared to current international nutritional guidelines (discussed above). Assessing micronutrient status from blood, or other accessible and non-invasive samples, is notoriously difficult in this context (130). Animal models of undernutrition could therefore be used to evaluate how different physiological features of undernutrition affect dietary nutrient requirements. The immunological effects of individual dietary components, such as staple foods consumed in LMIC but understudied in experimental nutrition (23), could also be systematically evaluated in animal models to formulate novel immunotherapeutic foods. Nonetheless, animals that are severely deficient in one nutrient but replete in all others, are unlikely to represent a good model for an acutely malnourished child, and future animal models should be developed to better re-capitulate the human undernourished state.

#### Model Microbes

Recurrent exposure to pathogenic microorganisms and an altered microbiome structure and function are characteristic of children at risk of undernutrition in LMIC. Microbiome-focused studies are at the forefront of translational research in malnutrition [extensively reviewed elsewhere; e.g., (13, 23)]. The predominant experimental approach has been to colonize mice with the fecal microbiome of children with SAM to demonstrate its causal role in weight loss and impaired nutrient metabolism (126), albeit reflecting the microbiome composition of individual children at a single timepoint. Innate immune function has not been explored in these models. Furthermore, in the gnotobiotic, germ-free and specific pathogen-free (SPF) animals used, microbial exposures are necessarily limited and defined. In contrast, children in LMIC begin to experience microbial exposures *in utero* and pathogen exposures continue throughout early life in response to mode of delivery, pattern of breastfeeding, local diets, and household water, sanitation and hygiene conditions [as recently reviewed (13)]; individual exposure histories may therefore have a critical role in shaping the interdependent development of gut, microbiome and innate immune system, and *vice versa*. For example, development of the gastrointestinal tract is severely compromised in mice that genetically lack TLR and IL-1 signaling molecules (131). An alternative approach to using gnotobiotic/germ-free/SPF animals has been to intensively treat conventionally-housed animals with antibiotics to ablate an established “healthy” microbiome and then re-colonize with the fecal microbiota of an undernourished child. However, this approach also has limitations since antibiotic effects on the microbiome are associated with heritable pro-inflammatory intestinal immune responses (132). In addition, a number of important factors impact the translational relevance of microbial colonization models of childhood undernutrition (13). Collecting child stool samples is non-invasive and stool reflects colonic luminal microbiota; however, the stool microbiome is qualitatively different to that of the small intestine (133), where most immune surveillance, antigen detection, pathogen challenge, and microbial translocation occurs (13). Human gastrointestinal microbes also vary in their ability to colonize other species, meaning that bacterial strain loss and/or quantitative differences in the resulting model microbiome are expected. Similarly, the behavior of gastrointestinal pathogens may differ across host species and thus alter the innate response generated in models of infection and undernutrition. Developing existing models of the undernourished microbiome would benefit from incorporating tandem assessment of innate and adaptive immune responses in the gut (and at other sites) and assessment of how undernutrition across the life-course affects colonization, infection and immune responses. Human cohort studies of longitudinal microbiome assemblies at different anatomical sites during undernutrition in LMIC are required to inform new and more physiologically relevant modifications to animal models.

#### Model Environments

Children frequently experience environmental exposures that influence immune development and such exposures may also modify the impact of undernutrition on immune cell function. These include: (i) Environmental toxin exposure, such as exposure to mycotoxins derived from molds that contaminate staple crops, which is extremely common amongst pregnant mothers and children in some settings (134). Several mycotoxins can modulate mammalian innate immune cell functions directly (135–137), or promote systemic inflammation by impacting gut barrier function (134, 138). (ii) Helminth infections are highly prevalent among children in LMIC and can directly drive undernutrition and distinct immune cell activation phenotypes, whilst also interacting with gastrointestinal commensals and pathogens (discussed above). (iii) Stress and chronic anxiety impair immune function, and can occur in caregivers and children in LMIC in association with, or as a direct consequence of, undernutrition (139, 140). (iv) Clinical interventions may have a distinct impact on immune function in undernourished vs. adequately-nourished children. As vaccination and treatment coverage increase in LMIC, understanding how undernutrition affects vaccine efficacy and the impact of antibiotics and anti-helminthics on microbial exposures and immune function is becoming increasingly relevant. For example, there is evidence that circulating vaccine-specific antibody levels are largely unaffected by undernutrition (45), but no studies have assessed the innate immune response to vaccination or accessory benefits of innate immune training by vaccines in undernutrition. Antibiotics, vitamin A and other micronutrient supplements are recommended for the management of children with SAM, which may all modulate infectious susceptibility. Furthermore, some antibiotics directly alter inflammation and innate immune cell function [e.g., cotrimoxazole (141)]. Animal models would provide an ideal way of characterizing the effects of these different environmental stressors during undernutrition, including the impact of epigenetic responses to these stimuli. Of particular interest would be the relative impact of multiple simultaneous stressors vs. individual environmental exposures, which are challenging to disaggregate in human cohort studies. Such models could inform translational studies trialing interventions to reduce exposure to stressors during early life.

## Conclusions

Existing studies have been critical to our understanding of innate immune cell function in childhood undernutrition, but considerable knowledge gaps remain. Studies among undernourished children demonstrate impairments in a range of innate immune responses, but the pathways underlying these defects are unclear. Experimental animal models provide a clearer picture of how individual features of the undernourished state (e.g., infections, micronutrient deficiencies, enteropathy) can drive changes in innate immune cell function, but do not recapitulate the multiple simultaneous immune challenges that are typical during childhood undernutrition in LMIC. It is increasingly apparent that novel therapeutic approaches are required to improve health outcomes for children with undernutrition, given the complex pathology that underlies wasting and stunting. Since infections are the leading cause of death (1), a better understanding of innate immune function could inform future tractable immune-targeted interventions for childhood undernutrition to reduce mortality and improve long-term health, growth and development.

## Author Contributions

CB wrote the manuscript with input and critical commentary from KJ and AP.

### Conflict of Interest Statement

The authors declare that the research was conducted in the absence of any commercial or financial relationships that could be construed as a potential conflict of interest.

## References

[1] Olofin I McDonald CM Ezzati M Flaxman S Black RE Fawzi WW. Associations of suboptimal growth with all-cause and cause-specific mortality in children under five years: a pooled analysis of ten prospective studies. PLoS ONE. (2013) 8:e64636. 10.1371/journal.pone.0064636 23734210 PMC3667136

[2] Black RE Victora CG Walker SP Bhutta ZA Christian P de Onis M. Maternal and child undernutrition and overweight in low-income and middle-income countries. Lancet. (2013) 382:427–51. 10.1016/S0140-6736(13)60937-X 23746772

[3] UNICEF, WHO, and W Bank UNICEF-WHO-The World Bank: Joint child Malnutrition Estimates — Levels and Trends – 2019 edition, (2019).

[4] Osgood-Zimmerman A Millear AI Stubbs RW Shields C Pickering BV Earl L. Mapping child growth failure in Africa between 2000 and 2015. Nature. (2018) 555:41. 10.1038/nature25760 29493591 PMC6346257

[5] Bourke CD Berkley JA Prendergast AJ. Immune dysfunction as a cause and consequence of malnutrition. Trends Immunol. (2016) 37:386–98. 10.1016/j.it.2016.04.003 27237815 PMC4889773

[6] Page AL de Rekeneire N Sayadi S Aberrane S Janssens AC Rieux C. Infections in children admitted with complicated severe acute malnutrition in Niger. PLoS ONE. (2013) 8:e68699. 10.1371/journal.pone.0068699 23874731 PMC3714292

[7] Berkley JA Ngari M Thitiri J Mwalekwa L Timbwa M Hamid F. Daily co-trimoxazole prophylaxis to prevent mortality in children with complicated severe acute malnutrition: a multicentre, double-blind, randomised placebo-controlled trial. Lancet Global Health. (2016) 4:e464–e473. 10.1016/S2214-109X(16)30096-1 27265353 PMC6132285

[8] Attia S Versloot CJ Voskuijl W van Vliet SJ Di Giovanni V Zhang L. Mortality in children with complicated severe acute malnutrition is related to intestinal and systemic inflammation: an observational cohort study. Am J Clin Nutr. (2016) 104:1441–9. 10.3945/ajcn.116.130518 27655441 PMC5081715

[9] Njunge J Gwela A Kibinge NK Ngari M Nyamako L Nyatichi E. Biomarkers of post-discharge mortality among children with complicated severe acute malnutrition. Sci Rep. (2019) 9:5981. 10.1038/s41598-019-42436-y 30979939 PMC6461700

[10] Versloot CJ Attia S Bourdon C Richardson SE Potani I Bandsma R. Intestinal pathogen clearance in children with severe acute malnutrition is unrelated to inpatient morbidity. Clin Nutr ESPEN. (2018) 24:109–13. 10.1016/j.clnesp.2018.01.004 29576347

[11] UNICEF, WHO, and W Bank, Levels and Trends in Child Malnutriton: UNICEF/WHO/World Bank Group Joint Child Malnutrition Estimates; Key findings of the 2017 edition (2017).

[12] Harper KM Mutasa M Prendergast AJ Humphrey J Manges AR. Environmental enteric dysfunction pathways and child stunting: a systematic review. PLoS Neglec Trop Dis. (2018) 12:e0006205. 10.1371/journal.pntd.0006205 29351288 PMC5792022

[13] Robertson RC Manges AR Finlay BB Prendergast AJ. The human microbiome and child growth; first 1000 days and beyond. Trends Microbiol. (2018). 27:131–47. 10.1016/j.tim.2018.09.008 30529020

[14] Golden M. The effects of malnutrition in the metabolism of children. Transac R Soc Trop Med Hygiene. (1988) 82:3–6. 10.1016/0035-9203(88)90245-3 3140444

[15] Semba RD Shardell M Trehan I Moaddel R Maleta KM Ordiz MI. Metabolic alterations in children with environmental enteric dysfunction. Sci Rep. (2016) 6:28009. 10.1038/srep28009 27294788 PMC4904796

[16] Prendergast AJ Rukobo S Chasekwa B Mutasa K Ntozini R Mbuya MNN. Stunting is characterized by chronic inflammation in zimbabwean infants. PLoS ONE. (2014) 9:e86928. 10.1371/journal.pone.0086928 24558364 PMC3928146

[17] Jones KDJ Hünten-Kirsch B Laving AMR Munyi CW Ngari M Mikusa J. Mesalazine in the initial management of severely acutely malnourished children with environmental enteric dysfunction: a pilot randomized controlled trial. BMC Med. (2014) 12:133. 10.1186/s12916-014-0133-2 25189855 PMC4243388

[18] Paul KH Muti M Chasekwa B Mbuya MN Madzima RC Humphrey JH. Complementary feeding messages that target cultural barriers enhance both the use of lipid-based nutrient supplements and underlying feeding practices to improve infant diets in rural Zimbabwe. Matern Child Nutr. (2012) 8:225–38. 10.1111/j.1740-8709.2010.00265.x 22405701 PMC6860737

[19] Prendergast AJ Humphrey JH. The stunting syndrome in developing countries. Paediatr Int Child Health. (2014) 34:250–65. 10.1179/2046905514Y.0000000158 25310000 PMC4232245

[20] Prentice AM Ward KA Goldberg GR Jarjou LM Moore SE Fulford AJ. Critical windows for nutritional interventions against stunting. Am J Clin Nutr. (2013) 97:911–8. 10.3945/ajcn.112.052332 23553163 PMC3628381

[21] Shattuck-Heidorn H Reiches MW Prentice AM Moore SE Ellison PT Energetics and the immune system: trade-offs associated with non-acute levels of CRP in adolescent Gambian girls. Evol Med Public Health. (2016) 2017:27–38. 10.1093/emph/eow034 PMC5381351 28003312

[22] Nairz M Haschka D Demetz E Weiss G. Iron at the interface of immunity and infection. Front Pharmacol. (2014) 5:00152. 10.3389/fphar.2014.00152 25076907 PMC4100575

[23] Barratt MJ Lebrilla C Shapiro H-Y Gordon JI. The gut microbiota, food science, and human nutrition: a timely marriage. Cell Host Microbe. (2017) 22:134–41. 10.1016/j.chom.2017.07.006 28799899 PMC5915309

[24] Netea MG Schlitzer A Placek K Joosten LAB Schultze JL. Innate and adaptive immune memory: an evolutionary continuum in the host's response to pathogens. Cell Host Microbe. (2019) 25:13–26. 10.1016/j.chom.2018.12.006 30629914

[25] Benn CS Netea MG Selin LK Aaby P. A small jab – a big effect: nonspecific immunomodulation by vaccines. Trends Immunol. (2013) 34:431–9. 10.1016/j.it.2013.04.004 23680130

[26] Arts RJ Blok BA van Crevel R Joosten LA Aaby P Benn CS. Vitamin A induces inhibitory histone methylation modifications and down-regulates trained immunity in human monocytes. J Leukocyte Biol. (2015) 98:129–36. 10.1189/jlb.6AB0914-416R 25934925

[27] Ordovas-Montanes J Dwyer DF Nyquist SK Buchheit KM Vukovic M Deb C. Allergic inflammatory memory in human respiratory epithelial progenitor cells. Nature. (2018) 560:649–54. 10.1038/s41586-018-0449-8 30135581 PMC6133715

[28] Cassone A. The case for an expanded concept of trained immunity. mBio. (2018) 9:e00570–18. 10.1128/mBio.00570-18 29789368 PMC5964355

[29] Naik S Larsen SB Gomez NC Alaverdyan K Sendoel A Yuan S. Inflammatory memory sensitizes skin epithelial stem cells to tissue damage. Nature. (2017) 550:475–80. 10.1038/nature24271 29045388 PMC5808576

[30] Delano MJ Ward PA. Sepsis-induced immune dysfunction: can immune therapies reduce mortality? J Clin Invest. (2016) 126:23–31. 10.1172/JCI82224 26727230 PMC4701539

[31] Grondman I Arts RJW Koch RM Leijte GP Gerretsen J Bruse N. Endotoxin-induced immunotolerance is associated with loss of monocyte metabolic plasticity and reduction of oxidative burst. J Leukoc Biol. (2019) 106:11–25. 10.1002/JLB.5HI0119-018R 31169935 PMC6852552

[32] Shalova IN Lim JY Chittezhath M Zinkernagel AS Beasley F Hernandez-Jimenez E. Human monocytes undergo functional re-programming during sepsis mediated by hypoxia-inducible factor-1alpha. Immunity. (2015) 42:484–98. 10.1016/j.immuni.2015.02.001 25746953

[33] van der Poll T van de Veerdonk FL Scicluna BP Netea MG. The immunopathology of sepsis and potential therapeutic targets. Nat Rev Immunol. (2017) 17:407. 10.1038/nri.2017.36 28436424

[34] Uchiyama R Kupkova K Shetty SJ Linford AS Pray-Grant MG Wagar LE. Histone H3 lysine 4 methylation signature associated with human undernutrition. Proc Natl Acad Sci USA. (2018). 115:E11264–E11273. 10.1073/pnas.1722125115 30420518 PMC6275549

[35] Lima AAM Leite ÁM Di Moura A Lima NL Soares AM Abreu CB. Determinant variables, enteric pathogen burden, gut function and immune-related inflammatory biomarkers associated with childhood malnutrition: a prospective case-control study in Northeastern Brazil. Pediatric Infec Dis J. (2017) 36:1177–85. 10.1097/INF.0000000000001569 28230705 PMC5568907

[36] Zhang L Voskuijl W Mouzaki M Groen AK Alexander J Bourdon C. Impaired bile acid homeostasis in children with severe acute malnutrition. PLoS One. (2016) 11:e0155143. 10.1371/journal.pone.0155143 27163928 PMC4862637

[37] Lee SE Stewart CP Schulze KJ Cole RN Wu LS Yager JD. The plasma proteome is associated with anthropometric status of undernourished nepalese school-aged children. J Nutr. (2017) 147:304–13. 10.3945/jn.116.243014 28148680 PMC5320403

[38] Guerrant RL Leite AM Pinkerton R Medeiros PHQS Cavalcante PA DeBoer M. Biomarkers of environmental enteropathy, inflammation, stunting, and impaired growth in children in Northeast Brazil. PLoS ONE. (2016) 11:e0158772. 10.1371/journal.pone.0158772 27690129 PMC5045163

[39] Kosek MN Team M-E. Causal pathways from enteropathogens to environmental enteropathy: findings from the MAL-ED birth cohort study. EBioMed. (2017) 18:109–17. 10.1016/j.ebiom.2017.02.024 28396264 PMC5405169

[40] Donowitz J Cook H Alam M Tofail F Kabir MM Colgate ER. Role of maternal health and infant inflammation in nutritional and neurodevelopmental outcomes of two-year-old Bangladeshi children. PLoS Negl Trop Dis. (2018) 12:e0006363. 10.1371/journal.pntd.0006363 29813057 PMC5993301

[41] Farràs M Chandwe K Mayneris-perxachs J Amadi B Louis-Auguste J Besa E. Characterizing the metabolic phenotype of intestinal villus blunting in Zambian children with severe acute malnutrition and persistent diarrhea. PLoS One. (2018) 13:e0192092. 10.1371/journal.pone.0192092 29499047 PMC5834158

[42] Delamare G Chandwe K Lee W Drake L Frost G Holmes E Health Outcomes in Undernutrition: The Role of Nutrients, Gut Dysfunction and the Gut Microbiome (HUNGer) Consortium: White Paper, (2019).

[43] Page AL de Rekeneire N Sayadi S Aberrane S Janssens AC Dehoux M. Diagnostic and prognostic value of procalcitonin and C-reactive protein in malnourished children. Pediatrics. (2014) 133:e363–70. 10.1542/peds.2013-2112 24446443

[44] Rytter MJ Babirekere-Iriso E Namusoke H Christensen VB Michaelsen KF Ritz C. Risk factors for death in children during inpatient treatment of severe acute malnutrition: a prospective cohort study. Am J Clin Nutr. (2017) 105:494–502. 10.3945/ajcn.116.140822 28031190

[45] Rytter MJ Kolte L Briend A Friis H Christensen VB The immune system in children with malnutrition–a systematic review. PLoS ONE. (2014) 9:e105017 10.1371/journal.pone.0105017 25153531 PMC4143239

[46] Morgan G. What, if any, is the effect of malnutrition on immunological competence? Lancet. (1997) 349:1693–5. 10.1016/S0140-6736(96)12038-9 9186397

[47] Qi R Huang Y-T Liu J-W Sun Y Sun X-F Han H-J. Global prevalence of asymptomatic norovirus infection: a meta-analysis. EClinicalMed. (2018) 2:50–8. 10.1016/j.eclinm.2018.09.001 31193628 PMC6537540

[48] Piriou E Asito AS Sumba PO Fiore N Middeldorp JM Moormann AM. Early age at time of primary Epstein-Barr virus infection results in poorly controlled viral infection in infants from Western Kenya: clues to the etiology of endemic Burkitt lymphoma. J Infec Dis. (2012) 205:906–13. 10.1093/infdis/jir872 22301635 PMC3282570

[49] Eibach D Krumkamp R Hahn A Sarpong N Adu-Sarkodie Y Leva A. Application of a multiplex PCR assay for the detection of gastrointestinal pathogens in a rural African setting. BMC Infec Dis. (2016) 16:150. 10.1186/s12879-016-1481-7 27080387 PMC4832549

[50] Marie C Ali A Chandwe K Petri WA Kelly P. Pathophysiology of environmental enteric dysfunction and its impact on oral vaccine efficacy. Mucosal Immunol. (2018) 11:1290–8. 10.1038/s41385-018-0036-1 29988114

[51] Fumagalli M Pozzoli U Cagliani R Comi GP Riva S Clerici M. Parasites represent a major selective force for interleukin genes and shape the genetic predisposition to autoimmune conditions. J Exp Med. (2009) 206:1395–408. 10.1084/jem.20082779 19468064 PMC2715056

[52] Ferrer-Admetlla A Bosch E Sikora M Marquès-Bonet T Ramírez-Soriano A Muntasell A. Balancing selection is the main force shaping the evolution of innate immunity genes. J Immunol. (2008) 181:1315–22. 10.4049/jimmunol.181.2.1315 18606686

[53] Duchene J Novitzky-Basso I Thiriot A Casanova-Acebes M Bianchini M Etheridge SL. Atypical chemokine receptor 1 on nucleated erythroid cells regulates hematopoiesis. Nat Immunol. (2017) 18:753–61. 10.1038/ni.3763 28553950 PMC5480598

[54] Kerac M Bunn J Chagaluka G Bahwere P Tomkins A Collins S. Follow-up of post-discharge growth and mortality after treatment for severe acute malnutrition (FuSAM Study): a prospective cohort study PLoS One. (2014) 9:e96030. 10.1371/journal.pone.0096030 24892281 PMC4043484

[55] Bwakura-Dangarembizi M Amadi B Bourke C Robertson R Mwapenya B Chandwe K. Health outcomes, pathogenesis and epidemiology of severe acute malnutrition (HOPE-SAM): rationale and methods of a longitudinal observational study. BMJ Open. (2018) 9:e023077. 10.1136/bmjopen-2018-023077 30782694 PMC6361330

[56] Prendergast AJ Berejena C Pimundu G Shonhai A Bwakura-Dangarembizi M Musiime V. Inflammatory biomarkers in HIV-infected children hospitalized for severe malnutrition in Uganda and Zimbabwe. AIDS. (2019) 33:1485–90. 10.1097/QAD.0000000000002231 31008797

[57] Douglas SD Schopfer K. Phagocyte function in protein-calorie malnutrition. Clin Exp Immunol. (1974) 17:121–8. 4143112 PMC1554055

[58] Jose DG Shelton M Tauro GP Belbin R Hosking CS. Deficiency of immunological and phagocytic function in aboriginal children with protein-calorie malnutrition. Med J Austr. (1975) 2:699–705. 813096 10.5694/j.1326-5377.1975.tb106221.x

[59] Ford GW Belbin R Jose DG Vorbach EA Kirke DK. Growth and immune function in aboriginal children during recovery from malnutrition and infection. Austral N Zealand J Med. (1976) 6:321–8. 10.1111/imj.1976.6.4.321 828048

[60] Keusch GT Urrutia JJ Guerrero O Castaneda G Smith H. Serum opsonic activity in acute protein-energy malnutrition. Bull World Health Organ. (1981) 59:923–929. 6802507 PMC2396131

[61] Shilotri PG. Hydrogen peroxide production by leukocytes in protein-calorie malnutrition. Clin Chim Acta Int J Clin Chem. (1976) 71:511–4. 10.1016/0009-8981(76)90103-0 822969

[62] Rosen EU Geefhuysen J Anderson R Joffe M Rabson AR. Leucocyte function in children with kwashiorkor. Arch Dis Childhood. (1975) 50:220–4. 10.1136/adc.50.3.220 1147655 PMC1544509

[63] Schopfer K Douglas SD. Neutrophil function in children with kwashiorkor. J Lab Clin Med. (1976) 88:450–61. 784886

[64] Shousha S Kamel K. Nitro blue tetrazolium test in children with kwashiorkor with a comment on the use of latex particles in the test. J Clin Pathol. (1972) 25:494–7. 10.1136/jcp.25.6.494 5043375 PMC477365

[65] Selvaraj RJ Bhat KS. Phagocytosis and leucocyte enzymes in protein-calorie malnutrition. Biochem. J. (1972) 127:255–9. 10.1042/bj1270255 4403728 PMC1178580

[66] Hughes SM Amadi B Mwiya M Nkamba H Tomkins A Goldblatt D. Dendritic cell anergy results from endotoxemia in severe malnutrition. J Immunol. (2009) 183:2818–26. 10.4049/jimmunol.0803518 19625645

[67] Singh VV Chauhan SK Rai R Kumar A Singh SM Rai G. Decreased pattern recognition receptor signaling, interferon-signature, and bactericidal/permeability-increasing protein gene expression in cord blood of term low birth weight human newborns. PLoS ONE. (2013) 8:e62845. 10.1371/journal.pone.0062845 23626859 PMC3633842

[68] Heikens GT Bunn J Amadi B Manary M Chhagan M Berkley JA. Case management of HIV-infected severely malnourished children: challenges in the area of highest prevalence. Lancet. (2008) 371:1305–7. 10.1016/S0140-6736(08)60565-6 18406865

[69] Amadi B Besa E Zyambo K Kaonga P Louis-Auguste J Chandwe K. Impaired barrier function and autoantibody generation in malnutrition enteropathy in zambia. EBioMed. (2017) 22:191–9. 10.1016/j.ebiom.2017.07.017 28750860 PMC5552244

[70] Welsh FKS Farmery SM MacLennan K Sheridan MB Barclay GR Guillou PJ. Gut barrier function in malnourished patients. Gut. (1998) 42:396–401. 10.1136/gut.42.3.396 9577348 PMC1727047

[71] Sturgeon JP Bourke CD Prendergast AJ. Children with noncritical infections have increased intestinal permeability, endotoxemia and altered innate immune responses. Pediatr Infec Dis J Online First. (2019) 38:741–8. 10.1097/INF.0000000000002311 30985520 PMC7614937

[72] Campbell DI Murch SH Elia M Sullivan PB Sanyang MS Jobarteh B. Chronic T cell-mediated enteropathy in rural west african children: relationship with nutritional status and small bowel function. Pediatric Res. (2003) 54:306. 10.1203/01.PDR.0000076666.16021.5E 12788978

[73] Liu J Bolick DT Kolling GL Fu Z Guerrant RL. Protein malnutrition impairs intestinal epithelial cell turnover, a potential mechanism of increased cryptosporidiosis in a murine model. Infec Immunity. (2016) 84:3542–9. 10.1128/IAI.00705-16 27736783 PMC5116730

[74] Ueno PM Ori,á RB Maier EA Guedes M Azevedo OGD Wu D. Alanyl-glutamine promotes intestinal epithelial cell homeostasis in vitro and in a murine model of weanling undernutrition. Am J Physiol Gastrointestinal Liver Physiol. (2011) 301:G612–22. 10.1152/ajpgi.00531.2010 21799183 PMC3191556

[75] Vlasova AN Paim FC Kandasamy S Alhamo MA Fischer DD Langel SN. Protein malnutrition modifies innate immunity and gene expression by intestinal epithelial cells and human rotavirus infection in neonatal gnotobiotic pigs. mSphere. (2017) 2:e00046–17. 10.1128/mSphere.00046-17 28261667 PMC5332602

[76] Brown EM Wlodarska M Willing BP Vonaesch P Han J Reynolds LA. Diet and specific microbial exposure trigger features of environmental enteropathy in a novel murine model. Nat Commun. (2015) 6:7806. 10.1038/ncomms8806 26241678 PMC4532793

[77] Yang WH Heithoff DM Aziz PV Sperandio M Nizet V Mahan MJ. Recurrent infection progressively disables host protection against intestinal inflammation. Science. (2017) 358:eaao5610. 10.1126/science.aao5610 29269445 PMC5824721

[78] D'Addio F La Rosa S Maestroni A Jung P Orsenigo E Ben Nasr M. Circulating IGF-I and IGFBP3 levels control human colonic stem cell function and are disrupted in diabetic enteropathy. Cell Stem Cell. (2015) 17:486–98. 10.1016/j.stem.2015.07.010 26431183 PMC4826279

[79] Liang S Guo X-K Ou J Huang R Xue Q Hu X. Nutrient sensing by the intestinal epithelium orchestrates mucosal antimicrobial defense via translational control of Hes1. Cell Host Microbe. (2019) 25:706–18.e7. 10.1016/j.chom.2019.03.012 31053533

[80] Soehnlein O Steffens S Hidalgo A Weber C. Neutrophils as protagonists and targets in chronic inflammation. Nat Rev Immunol. (2017) 17:248. 10.1038/nri.2017.10 28287106

[81] Kumar V Bimal S Singh SK Chaudhary R Das S Lal C. Leishmania donovani: dynamics of L. donovani evasion of innate immune cell attack due to malnutrition in visceral leishmaniasis. Nutrition. (2014) 30:449–58. 10.1016/j.nut.2013.10.003 24607302

[82] Vasquez-Garibay E Campollo-Rivas O Romero-Velarde E Mendez-Estrada C Garcia-Iglesias T Alvizo-Mora JG. Effect of renutrition on natural and cell-mediated immune response in infants with severe malnutrition. J Pediatric Gastroenterol Nutr. (2002) 34:296–301. 10.1097/00005176-200203000-00015 11964957

[83] Takele Y Adem E Getahun M Tajebe F Kiflie A Hailu A. Malnutrition in Healthy individuals results in increased mixed cytokine profiles, altered neutrophil subsets and function. PLoS ONE. (2016) 11:e0157919. 10.1371/journal.pone.0157919 27548305 PMC4993519

[84] Youinou PY Garre MA Menez JF Boles JM Morin JF Pennec Y. Folic acid deficiency and neutrophil dysfunction. Am J Med. (1982) 73:652–7. 10.1016/0002-9343(82)90406-5 6814250

[85] Nayak KC Sethi AS Aggarwal TD Chadda VS Kumar KK. Bactericidal power of neutrophils in protein calorie malnutrition. Ind J Pediatrics. (1989) 56:371–7. 10.1007/BF02722303 2509345

[86] Gause WC Wynn TA Allen JE. Type 2 immunity and wound healing: evolutionary refinement of adaptive immunity by helminths. Nat Rev Immunol. (2013) 13:607–14. 10.1038/nri3476 23827958 PMC3789590

[87] Bourke CD Maizels RM Mutapi F. Acquired immune heterogeneity and its sources in human helminth infection. Parasitology. (2011) 138:139–59. 10.1017/S0031182010001216 20946693 PMC3021922

[88] Yap P Utzinger J Hattendorf J Steinmann P. Influence of nutrition on infection and re-infection with soil-transmitted helminths: a systematic review. Parasites Vectors. (2014) 7:229. 10.1186/1756-3305-7-229 24885622 PMC4032457

[89] Vila CC Saracino MP Falduto GH Calcagno MA Venturiello SM Pallaro AN. Protein malnutrition impairs the immune control of Trichinella spiralis infection. Nutrition. (2019) 60:161–9. 10.1016/j.nut.2018.10.024 30599460

[90] Lu H Xie RD Lin R Zhang C Xiao XJ Li LJ. Vitamin D-deficiency induces eosinophil spontaneous activation. Cell Immunol. (2017) 322:56–63. 10.1016/j.cellimm.2017.10.003 29050663

[91] Silverberg JI. Association between childhood atopic dermatitis, malnutrition, and low bone mineral density: a US population-based study. Pediatric Allergy Immunol. Offic Publication Eur Soc Pediatric Allergy Immunol. (2015) 26:54–61. 10.1111/pai.12315 25443466

[92] Takemoto S Yamamoto A Tomonaga S Funaba M Matsui T. Magnesium deficiency induces the emergence of mast cells in the liver of rats. J Nutr Sci Vitaminol. (2013) 59:560–3. 10.3177/jnsv.59.560 24477254

[93] Faivre V Lukaszewicz AC Alves A Charron D Payen D Haziot A. Accelerated in vitro differentiation of blood monocytes into dendritic cells in human sepsis. Clin Exp Immunol. (2007) 147:426–39. 10.1111/j.1365-2249.2006.03287.x 17302891 PMC1810505

[94] Leentjens J Kox M Koch RM Preijers F Joosten LA van der Hoeven JG. (2012). Reversal of immunoparalysis in humans *in vivo*. Am J Respir Crit Care Med. 186:838–45. 10.1164/rccm.201204-0645OC 22822024

[95] Patel AA Zhang Y Fullerton JN Boelen L Rongvaux A Maini AA. The fate and lifespan of human monocyte subsets in steady state and systemic inflammation. J Exp Med. (2017) 214:1913–23. 10.1084/jem.20170355 28606987 PMC5502436

[96] Wong KL Yeap WH Tai JJ Ong SM Dang TM Wong SC. The three human monocyte subsets: implications for health and disease. Immunol Res. (2012) 53:41–57. 10.1007/s12026-012-8297-3 22430559

[97] Peixoto Paes-Silva R Correia de Macedo EM Oliveira Tomiya MT Machado Barbosa de Castro CM Immune response of severe malnutrition children treated according to the protocol of the World Health Organization. Nutr Hospital. (2015) 32:638–44. 10.3305/nh.2015.32.2.9048 26268093

[98] Lotfy OA Saleh WA el-Barbari M. A study of some changes of cell-mediated immunity in protein energy malnutrition. J Egyp Soc Parasitol. (1998) 28:413–28. 9707671

[99] Niiya T Akbar SM Yoshida O Miyake T Matsuura B Murakami H. Impaired dendritic cell function resulting from chronic undernutrition disrupts the antigen-specific immune response in mice. J Nutr. (2007) 137:671–5. 10.1093/jn/137.3.671 17311958

[100] Hillyer LM Woodward B. Acutely malnourished weanling mice administered Flt3 ligand can support a cell-mediated inflammatory response. Cytokine. (2019) 113:39–49. 10.1016/j.cyto.2018.06.004 30539781

[101] Ibrahim M Barnes J Osorio E Anstead G Jimenez FJ Melby P. Deficiency of lymph node-resident Dendritic Cells (DCs) and dysregulation of DC chemoattractants in a malnourished mouse model of leishmania donovani infection. Infect Immun. (2014) 82:3098–112. 10.1128/IAI.01778-14 24818662 PMC4136237

[102] Epelman S Kory Lavine J Gwendalyn Randolph J. Origin and functions of tissue macrophages. Immunity. (2014) 41:21–35. 10.1016/j.immuni.2014.06.013 25035951 PMC4470379

[103] Ibrahim MK Zambruni M Melby CL Melby PC. Impact of childhood malnutrition on host defense and infection. Clin Microbiol Rev. (2017) 30:919–71. 10.1128/CMR.00119-16 28768707 PMC5608884

[104] Morais NGD Costa TBD Ferreira de Lima LF Basílio DDS Morais NNGD Paiva Cavalcanti MD. Impact of neonatal malnutrition on expression TLR-9, NF-kB and cytokines of macrophages infected in vitro with methicillin resistant Staphylococcus aureus. Microb Pathogen. (2019) 132:254–60. 10.1016/j.micpath.2019.05.009 31075429

[105] Kundu R Theodoraki A Haas CT Zhang Y Chain B Kriston-Vizi J. Cell-type-specific modulation of innate immune signalling by vitamin D in human mononuclear phagocytes. Immunology. (2017) 150:55–63. 10.1111/imm.12669 27608289 PMC5167305

[106] Desai A Smith LE Mbuya MN Chigumira A Fundira D Tavengwa NV. The SHINE trial infant feeding intervention: pilot study of effects on maternal learning and infant diet quality in rural zimbabwe. Clin Infect Dis. (2015) 61:S710–5. 10.1093/cid/civ846 26602298 PMC4657591

[107] Najera-Medina O Valencia-Chavarria F Cortes-Bejar C Palacios-Martinez M Rodriguez-Lopez CP Gonzalez-Torres MC. Infected malnourished children displayed changes in early activation and lymphocyte subpopulations. Acta paediatrica. (2017) 106:1499–506. 10.1111/apa.13930 28520183

[108] Salimonu LS Ojo-Amaize E Williams AIO Johnson AOK Cooke AR Adekunle FA. Depressed natural killer cell activity in children with protein-calorie malnutrition. Clin Immunol Immunopathol. (1982) 24:1–7. 10.1016/0090-1229(82)90082-4 6179671

[109] Salimonu LS Ojo-Amaize E Johnson AO Laditan AA Akinwolere OA Wigzell H. Depressed natural killer cell activity in children with protein–calorie malnutrition. II Correction of the impaired activity after nutritional recovery. Cell Immunol. (1983) 82:210–5. 10.1016/0008-8749(83)90154-5 6416685

[110] Gilchrist JJ Rautanen A Fairfax B Mills TC Naranbhai V Trochet H. Risk of nontyphoidal Salmonella bacteraemia in African children is modified by STAT4. (2018) 9:1014. 10.1038/s41467-017-02398-z 29523850 PMC5844948

[111] Di Francesco A Di Germanio C Bernier M de Cabo R. A time to fast. Science. (2018) 362:770–5. 10.1126/science.aau2095 30442801 PMC8504313

[112] Clinthorne JF Beli E Duriancik DM Gardner EM. NK cell maturation and function in C57BL/6 mice are altered by caloric restriction. J Immunol. (2013) 190:712–22. 10.4049/jimmunol.1201837 23241894 PMC4080414

[113] Ritz BW Aktan I Nogusa S Gardner EM. Energy restriction impairs natural killer cell function and increases the severity of influenza infection in young adult male C57BL/6 mice. J Nutr. (2008) 138:2269–75. 10.3945/jn.108.093633 18936230 PMC2635521

[114] Clinthorne JF Adams DJ Fenton JI Ritz BW Gardner EM. Short-Term Re-feeding of previously energy-restricted C57BL/6 male mice restores body weight and body fat and attenuates the decline in natural killer cell function after primary influenza infection. J Nutr. (2010) 140:1495–501. 10.3945/jn.110.122408 20534876 PMC2903303

[115] Eberl G Colonna M Di Santo JP McKenzie ANJ. Innate lymphoid cells: a new paradigm in immunology. Science. (2015) 348:aaa6566. 10.1126/science.aaa6566 25999512 PMC5658207

[116] Wilhelm C Kharabi Masouleh S Kazakov A. Metabolic regulation of innate lymphoid cell-mediated tissue protection-linking the nutritional state to barrier immunity. Front Immunol. (2017) 8:1742–1742. 10.3389/fimmu.2017.01742 29375541 PMC5770634

[117] Spencer SP Wilhelm C Yang Q Hall JA Bouladoux N Boyd A. Adaptation of innate lymphoid cells to a micronutrient deficiency promotes type 2 barrier immunity. Science. (2014) 343:432–7. 10.1126/science.1247606 24458645 PMC4313730

[118] Goverse G Labao-Almeida C Ferreira M Molenaar R Wahlen S Konijn T. Vitamin A controls the presence of RORgamma+ innate lymphoid cells and lymphoid tissue in the small intestine. J Immunol. 196:5148–55. 10.4049/jimmunol.1501106 27183576

[119] Li S Bostick JW Ye J Qiu J Zhang B Urban JFJr. Aryl hydrocarbon receptor signaling cell intrinsically inhibits intestinal group 2 innate lymphoid cell function. Immunity. (2018) 49:915–28.e5. 10.1016/j.immuni.2018.09.015 30446384 PMC6249058

[120] Wilhelm C Harrison OJ Schmitt V Pelletier M Spencer SP Urban JFJr. Critical role of fatty acid metabolism in ILC2-mediated barrier protection during malnutrition and helminth infection. J Exp Med. (2016) 213:1409–18. 10.1084/jem.20151448 27432938 PMC4986525

[121] Spencer SP Belkaid Y. Dietary and commensal derived nutrients: shaping mucosal and systemic immunity. Curr Opin Immunol. (2012) 24:379–84. 10.1016/j.coi.2012.07.006 22857854 PMC3431603

[122] Di Giovanni V Bourdon C Wang DX Seshadri S Senga E Versloot CJ. Metabolomic changes in serum of children with different clinical diagnoses of malnutrition. J Nutr. (2016) 146:2436–44. 10.3945/jn.116.239145 27807038 PMC5118769

[123] Saleh M Elson CO. Experimental inflammatory bowel disease: insights into the host-microbiota dialog. Immunity. (2011) 34:293–302. 10.1016/j.immuni.2011.03.008 21435584 PMC3108903

[124] Morlese JF Forrester T Del Rosario M Frazer M Jahoor F. Transferrin kinetics are altered in children with severe protein-energy malnutrition. J Nutr. (1997) 127:1469–74. 10.1093/jn/127.8.1469 9237939

[125] Hassanein E-SA Assem HM Rezk MM El-Maghraby RM Study of plasma albumin, transferrin, and fibronectin in children with mild to moderate protein-energy malnutrition. J Trop Pediatr. (1998) 44:362–5. 10.1093/tropej/44.6.362 9972082

[126] Smith MI Yatsunenko T Manary MJ Trehan I Mkakosya R Cheng J. Gut microbiomes of malawian twin pairs discordant for kwashiorkor. Science. (2013) 339:548–54. 10.1126/science.1229000 23363771 PMC3667500

[127] Wagner VE Dey N Guruge J Hsiao A Ahern PP Semenkovich NP. Effects of a gut pathobiont in a gnotobiotic mouse model of childhood undernutrition. Sci Transl Med. (2016) 8:366ra164. 10.1126/scitranslmed.aah4669 27881825 PMC5152673

[128] Mark Charbonneau R O'Donnell D Laura Blanton V Sarah Totten M Jasmine Davis CC Michael Barratt J. Sialylated milk oligosaccharides promote microbiota-dependent growth in models of infant undernutrition. Cell. (2016) 164:859–71. 10.1016/j.cell.2016.01.024 26898329 PMC4793393

[129] Coates JC Colaiezzi BA Bell W Charrondiere UR Leclercq C. Overcoming dietary assessment challenges in low-income countries: technological solutions proposed by the International Dietary Data Expansion (INDDEX) Project. Nutrients. (2017) 9:289. 10.3390/nu9030289 28300759 PMC5372952

[130] Dao DT Anez-Bustillos L Cho BS Li Z Puder M Gura KM. Assessment of micronutrient status in critically Ill children: challenges and opportunities. Nutrients. (2017) 9:1185. 10.3390/nu9111185 29143766 PMC5707657

[131] Rakoff-Nahoum S Kong Y Kleinstein SH Subramanian S Ahern PP Gordon JI. Analysis of gene-environment interactions in postnatal development of the mammalian intestine. Proc Natl Acad Sci USA. (2015) 112:1929–36. 10.1073/pnas.1424886112 25691701 PMC4343130

[132] Schulfer AF Battaglia T Alvarez Y Bijnens L Ruiz VE Ho M. Intergenerational transfer of antibiotic-perturbed microbiota enhances colitis in susceptible mice. Nat Microbiol. (2018) 3:234–42. 10.1038/s41564-017-0075-5 29180726 PMC5780248

[133] Yasuda K Oh K Ren B Tickle TL Franzosa EA Wachtman LM. Biogeography of the intestinal mucosal and lumenal microbiome in the rhesus macaque. Cell Host Microbe. (2015) 17:385–91. 10.1016/j.chom.2015.01.015 25732063 PMC4369771

[134] Smith LE Prendergast AJ Turner PC Mbuya MNN Mutasa K Kembo G. The potential role of mycotoxins as a contributorto stunting in the SHINE trial. Clin Infec Dis. (2015) 61(Suppl. 7):S733–7. 10.1093/cid/civ849 26602301 PMC4657594

[135] Marin DE Taranu I Burlacu R Tudor DS. Effects of zearalenone and its derivatives on the innate immune response of swine. Toxicon. (2010) 56:956–63. 10.1016/j.toxicon.2010.06.020 20615424

[136] Mehrzad J Klein G Kamphues J Wolf P Grabowski N Schuberth HJ *In vitro* effects of very low levels of aflatoxin B(1) on free radicals production and bactericidal activity of bovine blood neutrophils. Vet Immunol Immunopathol. (2011) 141:16–25. 10.1016/j.vetimm.2011.01.010 21377741

[137] Vandenbroucke V Croubels S Verbrugghe E Boyen F De Backer P Ducatelle R. The mycotoxin deoxynivalenol promotes uptake of Salmonella Typhimurium in porcine macrophages, associated with ERK1/2 induced cytoskeleton reorganization. Vet Res. (2009) 40:64. 10.1051/vetres/2009045 19674540

[138] Bol-Schoenmakers M Braber S Akbari P de Graaff P van Roest M Kruijssen L. The mycotoxin deoxynivalenol facilitates allergic sensitization to whey in mice. Mucosal Immunol. (2016) 9:1477–86. 10.1038/mi.2016.13 26883726

[139] Dhabhar FS. The short-term stress response – Mother nature's mechanism for enhancing protection and performance under conditions of threat, challenge, and opportunity. Front Neuroendocrinol. (2018) 49:175–92. 10.1016/j.yfrne.2018.03.004 29596867 PMC5964013

[140] Matare CR Mbuya MNN Pelto G Dickin KL Stoltzfus RJ. Assessing maternal capabilities in the SHINE trial: highlighting a hidden link in the causal pathway to child health. Clin Infect Dis. (2015) 61(Suppl. 7):S745–51. 10.1093/cid/civ851 26602303 PMC4657596

[141] Bourke CD Gough EK Pimundu G Shonhai A Berejena C Terry L. Cotrimoxazole reduces systemic inflammation in HIV infection by altering the gut microbiome and immune activation. Sci Transl Med. (2019) 11:eaav0537. 10.1126/scitranslmed.aav0537 30944164 PMC6783302

